# Seroprevalence of porcine reproductive and respiratory syndrome virus on swine farms in a tropical country of the Middle Americas: the case of Costa Rica

**DOI:** 10.1007/s11250-021-02799-9

**Published:** 2021-08-18

**Authors:** Ronald Meléndez, Mónica Guzmán, Carlos Jiménez, Marta Piche, Emily Jiménez, Bernal León, Juan M. Cordero, Lisbeth Ramirez-Carvajal, Alberto Uribe, Arie Van Nes, Arjan Stegeman, Hans Vernooij, Juan José Romero-Zúñiga

**Affiliations:** 1 Department of Farm Animal Health, University of Utrecht, Utrecht, the Netherlands; 2 School of Veterinary Medicine (EMV), Universidad Nacional, Heredia, Costa Rica; 3 Ministry of Livestock and Agriculture, Heredia, Costa Rica; 4 Department of Virology, School of Veterinary Medicine, Universidad Nacional, Heredia, Costa Rica; 5 Faryvet Company, Heredia, Costa Rica; 6 Boehringer Ingelheim, Guadalajara, Jalisco México

**Keywords:** Epidemiology, Prevalence, PRRS, Reproduction, Serological diagnosis, Swine production, Virus

## Abstract

Porcine reproductive and respiratory syndrome virus (PRRSV) causes significant economic losses to the swine industry worldwide. Little is known regarding the epidemiology of this infection in tropical countries. To address this problem in Costa Rica, a seroepidemiological study was carried out in two phases. In the first phase, a pilot study was conducted in nine farms with the clinical diagnosis of PRRSV. In total, 265 pig serum samples were collected from animals ranging in age from 1 to 15 weeks of age. This study aimed to establish the duration of maternal immunity in piglets, to identify the period of viremia, and to determine when seroconversion occurs. In the second phase, a cross-sectional serology study was performed on a representative sample of the Costa Rican national herds in the second phase. The twenty-five selected farms represent all provinces and were classified according to herd size (100 to 2000 sows). In each farm, pigs aged 8, 10, and 12 weeks were sampled, as well as gilts based on the pilot study. In total 1281 pigs were sampled across all 25 farms. The aim of the cross-sectional study was to quantify the seroprevalence of PRRSV in Costa Rican pig farms and to describe its geographical distribution in this tropical country. The prevalence of positive farms was 44% (11/25), and these farms were located in six of the seven provinces of Costa Rica. Overall, 58% (344/596) of the pigs were seropositive to PRRSV. The age of the pigs and the ecozone where farms were located were significantly related with PRRSV seroprevalence in animals and herds, respectively.

## Introduction

Porcine reproductive and respiratory syndrome virus (PRRSV) is one of the most important pig pathogens worldwide from an economic perspective. Holtkamp et al. (2010) calculated the costs associated with this disease to be around 663 million USD/year in the USA. In Europe, average losses related to PRRSV outbreaks were estimated to be around two piglets per sow per year (Nieuwenhuis et al., 2012).

As climate may influence the virus spread within and between farms, it is difficult to infer the occurrence and distribution of PRRSV in a tropical country from observations in countries with a more moderate climate. In the past, some studies have been carried out to assess the PRRSV infection prevalence in Central America, México (Morilla et al., 2003), Colombia (Mogollón et al., 2006), Venezuela (Diaz, 2006), and the Dominican Republic,(Ventura et al., 2013), but there are no recent reports about the disease. This also holds for Costa Rica, a tropical country located in Central America between Nicaragua and Panama, where PRRSV was first detected in 1996 but which has received little attention since.

Holdridge (1987) has identified various climate zones (ecozones) in Costa Rica: moist-low mountain forest, moist-pre mountain forest, very moist-low mountain forest, very moist-pre mountain forest, very wet tropical forest, and rainy-low mountain forest. This research aimed to assess the seroprevalence of PRRSV in pig farms of Costa Rica and to estimate its association with the age of the pigs, farm size, geolocation, distance, time, and ecozones.

According to the Livestock National Census (INEC, 2014, 2015), there are 14,600 pig holders in Costa Rica, but most of them have backyard farms. There are only around 150 commercial pig farms, and these farms produce 80% of the country’s pork. The total number of sows in Costa Rica is approximately 39,000, and the number of pigs slaughtered per year rounds 780,000.

## Material and methods

### Sample size

In the first phase, a total of 260 pigs (1 to 15 weeks of age) from 9 highly PRRSV virus infection-suspected farms were sampled. All samples were tested in parallel with a commercial enzyme-linked immunoassay (IDEXX Laboratories: 100.0% Se, 99.7% Sp) and endpoint PCR (Zorzetto- Fernandez, 2016).

The second phase, a serological analysis was performed using the IDEXX PRRS X3 kit (cat. 99–40,959). Only 87 out of all of the 150 pig commercial farrow-to-finish farms were affiliated to the National Chamber of Pig Farmers. These 87 farms were classified as large (> 500 sows, n = 14), medium-sized (200 to 500 sows, n = 62), or small (< 200 sows, n = 11). Twenty-five of these farms were selected using stratified sampling. Vaccination against PRRSV has never been applied on these farms.

The sample size to determine presence of infection in each farm was based on 5% within-herd prevalence (95% confidence level), resulting in 50 to 60 samples, depending on herd size (Cannon et al., 1982). We sampled pigs of 8, 10, and 12 weeks of age, as well as gilts. A total of 450 blood samples were obtained from the eight large farms, 550 from 12 medium farms, and 278 from 5 small farms. The starting point for the distance and time determinations was the Juan Santamaria International Airport, located in the middle of the central zone of the country (Alajuela). Based on this, distances and journey length (time determinations) from each farm to the designated starting point were estimated and retrieved using Google Maps.

### Laboratory testing

#### Blood samples analysis

The blood samples collected were placed in portable coolers that maintained refrigeration temperatures (4 °C) and were transported to the laboratory within 8 h. Once in the laboratory, the samples were centrifuged for 10 min at 14,000 rpm. The sera were separated and stored in 1.5 ml vials (Eppendorf) at − 80 °C until analysis.

#### Determination of the serological status of the animals

A commercial ELISA with reported 100.0% sensitivity and 99.7% specificity was used following the manufacturer’s instructions (IDEXX PRRS 3XR Ab ELISA; IDEXX Laboratories Inc. West brook, Maine, USA). This assay uses a specific epitope of the PRRSV nucleocapsid for testing the presence of antibodies against PRRSV-1 and PRRSV-2.

### Data capture and editing

**Fig. 1:**
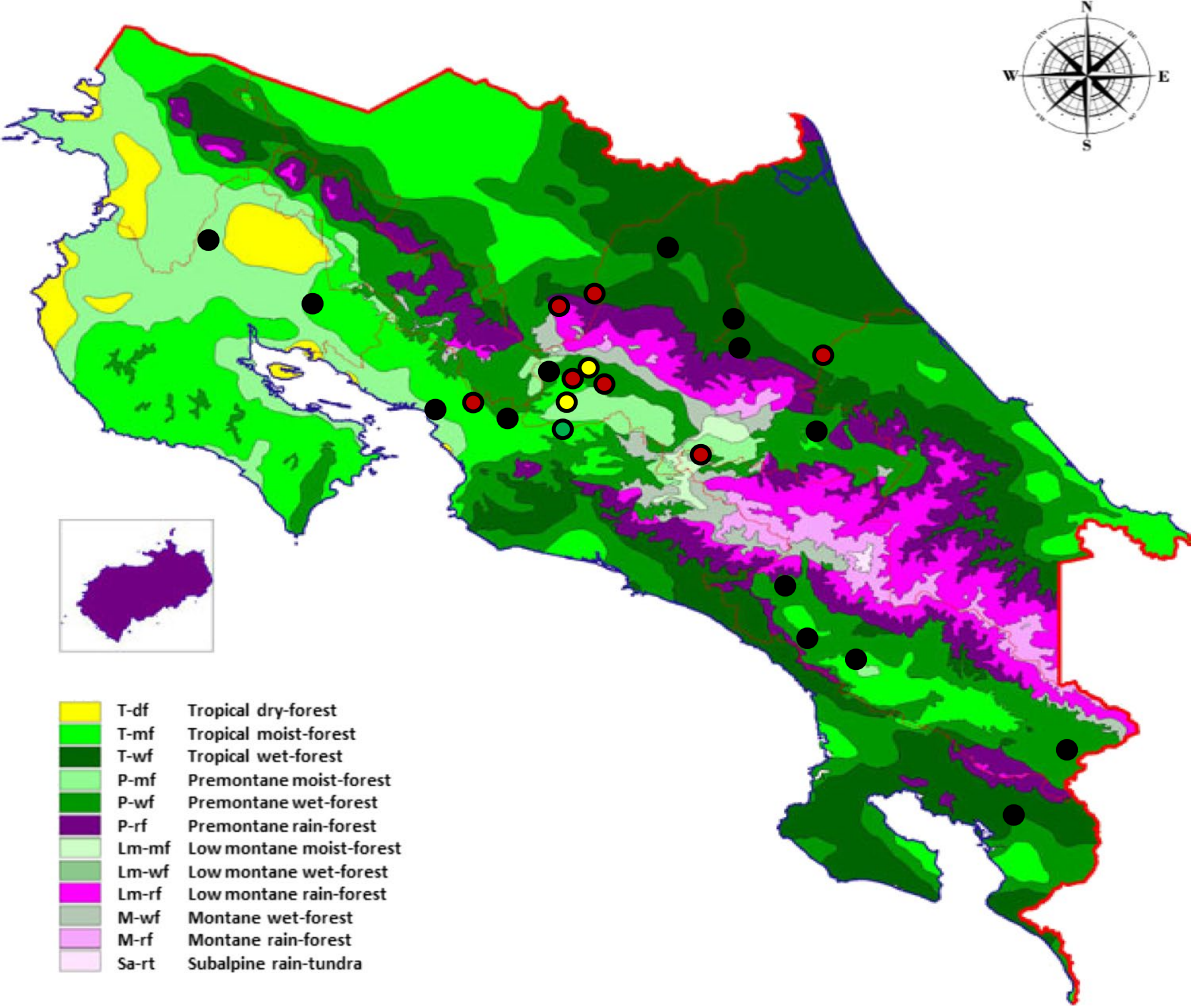
Within-herd seroprevalence of 25 farms depicted in a Costa Rican’s life ecozone map (Holdridge, 1971). The within-herd seroprevalence is depicted by spots: red = high (> 30); yellow = medium (15–30); green = (< 15); black = (0)

The geographical location of every farm included in this study was obtained from the “SIREA reconoce los esfuerzos ambientales de las instituciones” (2016) and depicted using ArcGIS® software (“ESRI 2011. ArcGIS Desktop: Release 10. Redlands, CA: Environmental Systems Research Institute” (2011)), firstly by province and also according to the Holdridge’s life zone classification (Holdridge, 1987). Costa Rica has twelve major life zones (Alfaro Murillo et al., 2013) defined more precisely twelve major life zones in Costa Rica (Fig. 1). Note that Holdridge also uses altitude, precipitation, and rain, evapotranspiration, and luminosity.

These life zones are defined and named according to the variation of temperature and precipitation in each area and the elevation above sea level. In Costa Rica, all forests are classified as tropical. Regarding humidity, they can be dry, moist, wet, and humid, whereas the elevational belt can be basal (0–700 masl); premontane (700–1400 masl); low montane (1400–2700 masl); montane (2400–3700 masl); subalpine (2400–3820 masl) (Table 1). In order to ease the description, the life zones were grouped and summarized from 12 ecozones into 6 ecozones: 1, P-wf = premontane wet forest; 2, T-mf = tropical moist forest; 3, P-mf = very humid premontane forest; 4, T-wf = tropical very humid forest; 5, P-rf = montane rain forest; 6, T-wf = tropical wet forest. The decision to switch from 12 to 6 ecozones was based on the pattern of temperature and rainfall. We do not have farms in the study with extreme weather conditions, neither hot nor cold.

**Table 1:** The altitude floor, temperatures, and altitude range of the most frequent life zones of Costa Rica according to Holdridge’s life zone classification

Altitude floor	Life zone	Temp (°C)	Altitude range (masl)
Basal (Coast influence)	Dry forest (T-df)	24	0–700
	Rain forest (T-mf)		
	Very humid forest (T-wf)		
Premontane (Coast influence)	Wet forest (P-mf)	24–18 °C	700–1400
	Very humid forest (P-wf)		
	Bosque pluvial (P-rf)		
Low montane	Rain forest (Lm-mf)	6–12 °C	1400–2700
	Very humid forest (Lm-wf)		
	Rain forest (Lm-rf)		
Montane	Very humid forest (M-wf)	6–12 °C	2400–3700
	Rain forest (M-rf)		
Alpine diving	Rain forest (Sa-)	3–6 °C	2400–3820

### Statistical analysis

First, a univariable logistic regression model was run using seropositivity at farm level as an outcome variable and farm size, age of the pigs, province, ecozone, distance, and traveling time to the farms since a reference point in the center of the country. Secondly, taking only the seropositive farms into account, a mixed effect logistic regression model was run at individual pig level (596 pigs). A stepwise backward model selection procedure was used based on the Akaike’s Information Criterion (AIC; smaller AIC is a better fitting model) to select the best fitting model. Statistical program SAS (*SAS/STAT ® 9.3 User’s Guide Introduction to Regression Procedures*, n.d.) was used to run the analyses.

## Results

The results obtained in the first phase about the dynamic of PRRSV indicate that maternal immunity is prolonged up to 3 weeks of life, that viremia occurs in the period between 10 and 15 weeks of life, and finally, that seroconversion occurs between weeks 10 and 15. In the second stage, a total of 11/25 (44.0%) farms were seropositive to PRRSV. The overall animal seroprevalence in this study was 26.9% (344/1281) 95% CI (24.5–29.4). The median within-herd seroprevalence in the seropositive farms was 58% (344/596), ranging from 1 to 100. In positive farms, seroprevalence increased with age; the seroprevalences were 39.4% in pigs of 8 weeks of age and 76.9% in breeders (Table 2). Geographically, all five farms located in the east-south region of the country tested negative. Farms located in the province of Guanacaste were all negative, while at least one of the sampled farms was positive in the other provinces. Moreover, the highest seroprevalence was observed in the life zones P-mf and P-wf (Table 3). Therefore, age and ecozones were significantly correlated with the seroprevalence in the statistical analysis (Table 3).

**Table 2:** Frequencies for positive results both at farm and animal level in swine farms of Costa Rica. Results are presents for all farms (25 farms, 1278 animals) and positive farms (11 farms, 596 animals)

Variable	Level	All farms				Positive farms (+ F_T =_11)	
		Farm (F_T =_ 25)		Animal (A_T_ = 1278)		Animal (+ A_T =_ 596)	
		+ f_t_ / F_T_	%	+ a_t_ / A_T_	%	a_t_ /A_T_	%
Farm size	Small	1/5	20.0	43/79	54.4	43/43	100.0
	Medium	7/12	58.3	190/715	26.6	190/350	54.3
	Large	3/8	37.5	111/487	22.8	111/203	54.7
Age	8 wks			54/310	17.4	54/137	9.4
	10 wks			68/295	23.1	68/139	48.9
	12 wks			82/311	26.4	82/138	59.4
	Replacem			140/365	38.4	140/182	76.9
Province	Alajuela	5/7	71.4	149/374	39.8	149/284	52.5
	Cartago	1/2	50.0	62/108	57.4	62/72	86.1
	Guanacaste	0/2	0.0	0/127	0.0		
	Heredia	1/3	33.3	43/90	47.8	43/45	95.6
	Limón	1/3	33.3	27/174	15.5	27/57	47.4
	Puntarenas	1/4	25.0	43/183	23.5	43/43	100.0
	San José	2/4	50.0	20/225	8.9	20/95	21.1
Ecozone	P-mf	5/10	50.0	157/515	30.5	157/267	58.8
	P-w	6/15	40.0	187/766	24.4	187/329	56.8
Distance from capital (km)	≤ 50	2 / 13	15.4	273/666	41.0	273/495	55.2
	> 55	9 / 12	75.0	71/615	11.5	71/101	70.3
Trav. time from capital (min.)	≤ 30	2 / 14	14.3	273 / 730	37.4	273/495	55.2
	> 50	9 / 11	81.8	71 / 551	12.9	71/101	70.3

**Table 3:** Summary of statistical analyses of univariate logistic regressions using seropositivity at farm level as the dependent variable (outcomes)

		Univariable					Multivariable			
Variable	Level	Estimate	OR	95% CI	P	Estimate		OR	95% CI	P
Farm size	Large	0.76	2.14	0.10–45.84	0.627					
	Medium	Ref								
Age	Replacement	3.23	25.24	11.54–55.18	< .001	3.27		26.22	1.17–4.92	< .001
	12	1.72	5.58	2.67–11.66		1.74		5.68	2.71–11.93	
	10	0.87	2.39	1.17–4.87		0.88		2.41	1.17–4.92	
	8	Ref								
Ecozone	Pm-f	2.83	17.09	2.20–132.60	0.007	3.24		25.51	1.78–366.55	0.017
	Pw-f	Ref								
Distance from capital (km)	≤ 50	0.26	1.30	0.07–23.36	0.859					
	> 55	Ref								
Time from capital (min)	≤ 50	-0.69	0.50	0.01–17.57	0.704					
	> 50	Ref								

## Discussion

The estimated prevalence of PRRSV in the national pig herd of Costa Rica was 44%. The median within-herd seroprevalence in the seropositive farms was 58%. The overall prevalence of PRRSV found in Canada was 37.1% (Magar & Larochelle, 2004) very similar in the USA (Bautista et al., 1993), México (Batista et al., 2004), and Spain (López‐Soria et al., 2010).

Of all the variables studied in the mixed-effect logistic regression model, only the age and the ecozones (Table 3) had a significant correlation with the seroprevalence. In the first stage, it was determined that the viremia begins at week seven and can extend until 15 weeks of age, while the production of antibodies starts at week 10. The link between the drop in maternal antibodies and the onset of viremia occurred in piglets at 10 weeks of age. The duration of viremia varies according to the PRRSV strain and the animal’s age (Pileri & Mateu, 2016).

Weaned piglets can become infected as their maternal antibodies disappear, and then they can continuously shed the virus for 3 to 8 weeks**.** As some reports showed, on most infected farms, most seroconversions already take place at 8 or 14 weeks of age (Lopez & Osorio, 2004). At the end of this period, most pigs (about 80–100%) are usually seropositive; however, the proportion of infected weaners varies among herds (Kwiecien et al., 2017) (Castro Mena, 2006). According to Evans et al. (2010), the seropositivity of young animals is indicative of the persistence of the virus in the population, while in adults, it could indicate a past exposure.

In our study, we found that ecozones are significantly related to the seroprevalence of PRRSV. Given the climatic conditions in Costa Rica, there is a significant variation in temperature during the day and night of up to 10 to 12 °C. Therefore, the relative humidity on rainy days increases from 60 to 85% humidity, which predisposes respiratory problems.

Some studies have been conducted to assess the temperature, humidity, and climate conditions of PRRSV, but the results were variable across countries (Arruda et al., 2015) (Tummaruk et al., 2015). One study suggested that the PRRSV was associated with temperature and relative humidity, while temperature had a more significant effect than relative humidity. Recently, it has been suggested that climatic factors (temperature, moisture, and land use) were associated with PRRSV outbreaks in the USA (Alkhamis et al., 2018). So, the Costa Rican climate conditions may promote PRRSV dissemination with warm temperatures and some humidity.

According to the geographical distribution of PRRSV in Costa Rica, the seropositive herds are mainly in the central zone and northern part of the country due to the high density of pig farms in this region. Notably, there are at least five slaughterhouses and ten feed plants (or mills) for animals around that area, and there is an active movement of pigs, food supply, and medicines. There is also the exchange of genetic material and semen to the farms, and then they may spread the virus throughout the national territory. However, the statistical analysis in our study showed that provinces, size of the farm, location, and distance were not related to the seroprevalence.

This study estimated the seroprevalence of PRRSV in pig farms in Costa Rica and found a significant correlation between the age of pigs and ecozones, suggesting that current control measures are not effective in eliminating and maintaining PRRSV freedom. This is the first time that ecozones have been linked to PRRSV seroprevalence in Costa Rica.

## Data Availability

The data sets analyzed during the current study are available from the corresponding author upon reasonable request.
